# Platelets Purification Is a Crucial Step for Transcriptomic Analysis

**DOI:** 10.3390/ijms23063100

**Published:** 2022-03-13

**Authors:** Mohamad Chebbo, Said Assou, Veronique Pantesco, Catherine Duez, Marie C. Alessi, Pascal Chanez, Delphine Gras

**Affiliations:** 1 Centre Cardiovasculaire et Nutrition, Aix-Marseille Université, INSERM, INRAE, 13005 Marseille, France; mohamad.chebbo@univ-amu.fr (M.C.); catherine.duez@inserm.fr (C.D.); marie-christine.alessi@univ-amu.fr (M.C.A.); pascal.chanez@univ-amu.fr (P.C.); 2 Institute for Regenerative Medicine and Biotherapy, Université de Montpellier, INSERM, Centre Hospitalier Universitaire de Montpellier, 34000 Montpellier, France; said.assou@inserm.fr (S.A.); veronique.pantesco@inserm.fr (V.P.); 3 Assistance Publique des Hôpitaux de Marseille, Hôpital de la Timone, Laboratoire d’hématologie, 13005 Marseille, France; 4 Assistance Publique des Hôpitaux de Marseille, Hôpital NORD, Clinique des Bronches, Allergies et Sommeil, 13015 Marseille, France

**Keywords:** platelets purification, transcriptome, leukocyte contamination

## Abstract

Platelets are small anucleate cells derived from the fragmentation of megakaryocytes and are involved in different biological processes especially hemostasis, thrombosis, and immune response. Despite their lack of nucleus, platelets contain a reservoir of megakaryocyte-derived RNAs and all the machinery useful for mRNA translation. Interestingly, platelet transcriptome was analyzed in health and diseases and led to the identification of disease-specific molecular signatures. Platelet contamination by leukocytes and erythrocytes during platelet purification is a major problem in transcriptomic analysis and the presence of few contaminants in platelet preparation could strongly alter transcriptome results. Since contaminant impacts on platelet transcriptome remains theoretical, we aimed to determine whether low leukocyte and erythrocyte contamination could cause great or only minor changes in platelet transcriptome. Using microarray technique, we compared the transcriptome of platelets from the same donor, purified by common centrifugation method or using magnetic microbeads to eliminate contaminating cells. We found that platelet transcriptome was greatly altered by contaminants, as the relative amount of 8274 transcripts was different between compared samples. We observed an increase of transcripts related to leukocytes and erythrocytes in platelet purified without microbeads, while platelet specific transcripts were falsely reduced. In conclusion, serious precautions should be taken during platelet purification process for transcriptomic analysis, in order to avoid platelets contamination and result alteration.

## 1. Introduction

For many years, platelets were considered as megakaryocyte-derived fragments exclusively involved in hemostasis and thrombosis. However, recent studies revealed that platelets may play a key role in several biological processes especially immune response, using their proteins and modulators pre-synthetized in megakaryocytes or internalized from blood by endocytosis [1]. Interestingly, these cells also contain a reservoir of RNAs derived from megakaryocytes and all the machinery useful for mRNA translation, allowing platelets to translate different mRNA onto proteins in response to various stimulants [2,3]. In addition, activated platelets can transfer their RNAs to recipient cells which result in alteration of gene expression levels in these latter [2,4]. These unexpected observations have attracted many researchers to study platelet transcriptome in different pathological conditions in order to better understand the significance and the potential of platelet RNA and to identify disease-specific molecular signatures. For instance, RNA sequence analysis of platelet from patient with gray platelet syndrome identified NBEAL2 as the causative gene of the disease [5]. In addition, platelets transcriptome was found altered in different viral infections such as HIV, influenza, and COVID-19 [6,7]. Interestingly, the interaction between tumor cells and platelets results in the transfer of tumor-associated biomolecules to platelets causing changes in platelet RNA and protein content. This process generates what is known as tumor-educated platelets (TEPs). It has been shown that transcriptome profiling of TEPs may be useful to distinguish between cancer patients and healthy donors and different methods for TEPs transcriptome analysis were developed for this purpose [8].

Considering the small amount of RNAs in platelets, a high number of these cells are usually necessary to obtain sufficient RNA quantity allowing the analysis of platelet transcriptome. A commonly used method for platelet rich plasma (PRP) preparation (hereafter called “classical method”) always leads to very low contamination by leukocytes and red blood cells [9]. However, in addition to the fact that platelets are used in relatively high number, it has been estimated that one leukocyte contains 1000 to 12,500 more RNA than platelet, suggesting that even very small contamination by leukocytes can strongly alter transcriptome results [10,11]. Moreover, despite their lack of nucleus, red blood cells also contain RNAs; thus, their presence in platelet preparation may result in the detection of erythrocyte transcripts [10]. Therefore, highly pure platelet preparation free of leukocytes and red blood cells contaminants is required for platelet transcriptome profiling. So far, different techniques for platelet purification have been used to eliminate contaminating cells including filters and magnetic microbeads. This leads to an increase in the cost of platelet purification process and a loss in the number of recovered platelets [9,12].

In this paper, we wanted to evaluate the real impact of contaminating cells on platelet transcriptome to determine whether low contamination could cause great or only minor changes in platelet gene expression profile. Using blood from same donor, platelets were collected either by the classical method or by the use of magnetic microbeads to eliminate contaminating leukocytes and red blood cells (hereafter called “microbead method”), then platelet transcriptome was analyzed by microarrays.

## 2. Results

### 2.1. Magnetic Microbeads Greatly Reduce Leukocyte and Erythrocyte Contamination

**Figure 1:**
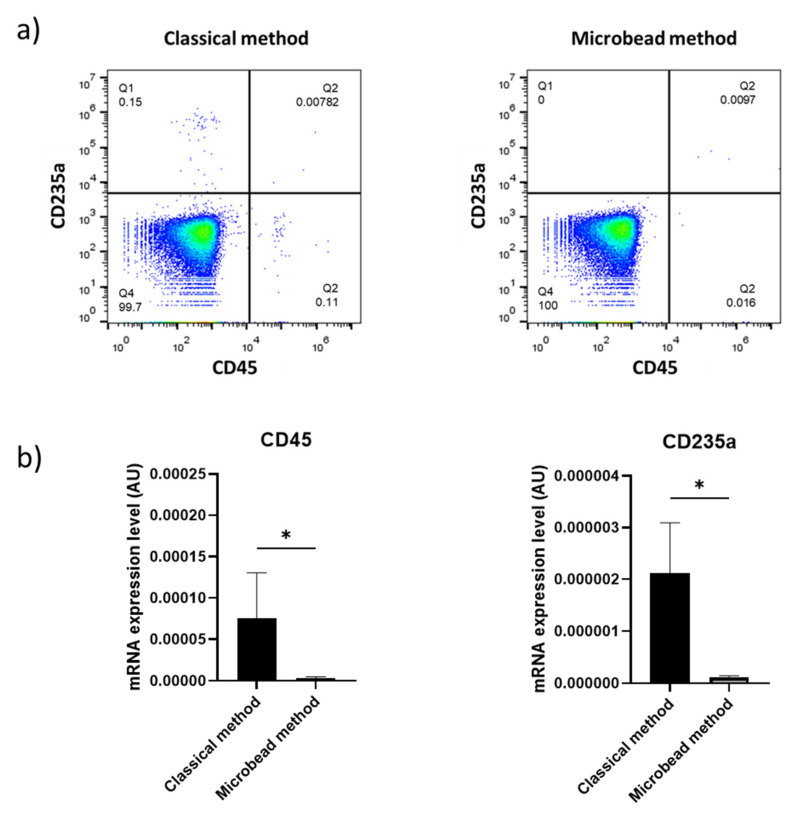
Platelet purity according to the purification method. (**a**) Flow cytometry analysis of platelets before and after the use of anti-CD45 and anti-CD235a magnetic microbeads to eliminate leukocytes and erythrocytes respectively. (**b**) RT-PCR quantification of CD45 and CD235a transcripts in total mRNA derived from platelets treated or not with magnetic microbeads. 18S was used as a reporter gene (*n* = 8). Results are expressed as relative expression compared to 18S in arbitrary unit (AU). Attached star * indicate a *p*-value < 0.05.

In order to confirm whether the use of magnetic microbeads was efficient to eliminate contaminating leukocytes and red blood cells from platelet preparations, we assessed the percentage of these contaminants in platelet preparation before and after the use of anti-CD45 and anti-CD235a microbeads by flow cytometry. We found that the percentage of leukocytes and red blood cells were strongly reduced after the use of magnetic microbeads (Figure 1a). These results were also confirmed by RT-PCR as the presence of CD45 and CD235a RNAs were substantially eliminated in microbead-treated samples (Figure 1b).

### 2.2. Leukocyte and Erythrocyte Contamination Strongly Alters Platelet Transcriptome Profile

**Figure 2:**
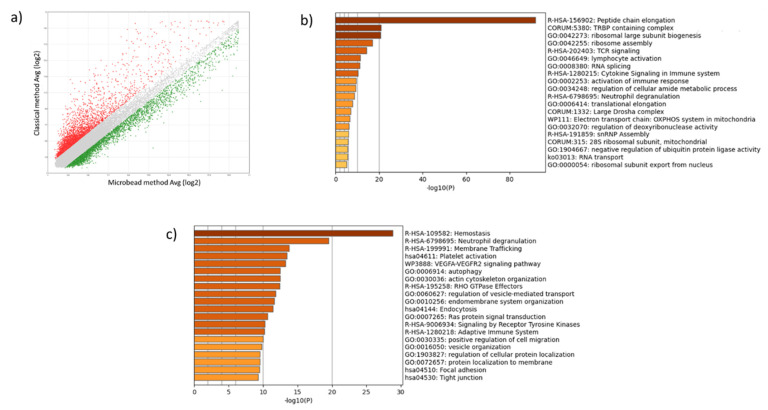
Differential presence of transcripts in platelets purified with or without microbeads. (**a**) Scatter plot showing the distribution of transcripts according to their relative level, presented as average log2 (RNA level). Gray dots indicate transcripts present at the same level in compared samples. Red dots indicate transcripts increased in platelets purified with classical method compared to those purified with microbeads, while green dots indicate decreased transcripts. (**b**,**c**) Metascape bar graph showing top enriched ontolog clusters in the lists of transcripts with increased and decreased relative amount (**b**,**c**, respectively) in platelets purified without microbeads.

Next, we compared by microarrays the transcriptome profile of platelets from the same donor purified by both the classical and the microbead methods. The scatter plot, where values are presented as average log2 (expression level), shows the great impact of contaminating leukocytes and erythrocytes on platelet transcriptome (Figure 2). Indeed, among the 135,750 genes analyzed, we found that the relative amount of 8274 transcripts were different between compared samples. The relative quantity of 3883 transcripts were increased in platelets preparation collected by the classical method compared to those collected using the microbead method, while the relative amount of 4391 transcripts were decreased (Figure 2a). GO annotation analysis on increased transcripts showed that terms related to ribosome are strongly enriched, in addition to other terms including TCR signaling, lymphocyte activation, cytokine signaling in immune system, and activation of immune response (Figure 2b). On the other side, GO annotation analysis on decreased transcripts showed that hemostasis and platelet activation terms were enriched (Figure 2c). Detailed data were presented in Figure S1 and Table S1 (see Supplementary Materials).

### 2.3. Increased Level of Specific Leukocyte and Erythrocyte Transcriptss in Contaminated Platelet Preparation

**Figure 3:**
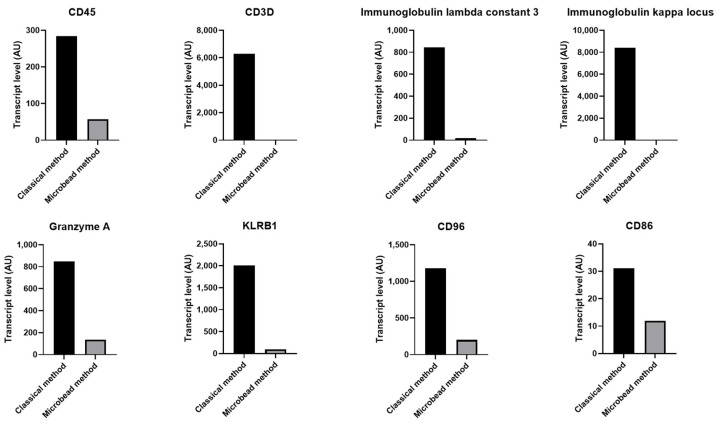
Stronger presence of transcripts related to leukocytes in platelets purified without magnetic microbeads. Histograms presenting transcript levels for a number of leukocyte genes, detected by microarray in platelet preparations purified with or without magnetic microbeads. Data were analyzed using RMA algorithm and TAC software. Different transcript levels with a fold change higher than 2 or lower than −2 were retained.

In concordance with PCR and flow cytometry results, microarray analysis shows that the relative amount of leukocyte specific transcript CD45 (PTPRC) was 56.91-fold higher in platelet collected from PRP without the use of magnetic microbeads compared with microbeads treated platelets (Figure 3). We also observed a higher relative quantity of transcripts specific for different leukocyte subsets such as T lymphocyte specific transcript CD3 and B lymphocyte transcripts including immunoglobulin heavy chain constant and variable regions, transcripts related to cytotoxic T cells and NK cells, e.g., KLRB1, CD94, and GZMA and antigen presenting cells, e.g., CD86 and MHC class II molecules (Figure 3). In addition, we observed an increased mRNA levels of immune receptors e.g., CXCR4, TLR10, IFNAR1 and 2, IFNGR2, IL7R, IL4R, and CCR7, and many other immune molecules (detailed in Supplementary Tables S2 and S5).

**Figure 4:**
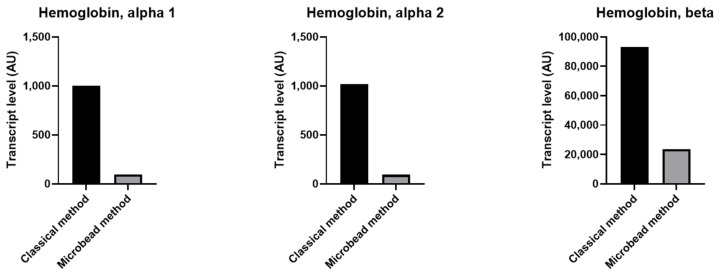
Increased relative level of transcripts related to erythrocytes in platelets purified without magnetic microbeads. Histograms presenting transcript levels for a number of erythrocyte genes, detected by microarray in platelet preparations purified with or without magnetic microbeads. Data were analyzed using RMA algorithm and TAC software. Different transcript levels with a fold change higher than 2 or lower than −2 were retained.

Although we could not detect the erythrocyte specific transcript GYPA (CD235a) in compared samples, we found an increased amount of hemoglobin alpha and beta transcripts in platelets collected by the classical method compared to those collected using magnetic microbeads (Figure 4 and Supplementary Tables S3 and S5), suggesting erythrocytes contamination.

### 2.4. Decreased Level of Transcripts Related to Platelets in Contaminated Platelet Preparation

**Figure 5:**
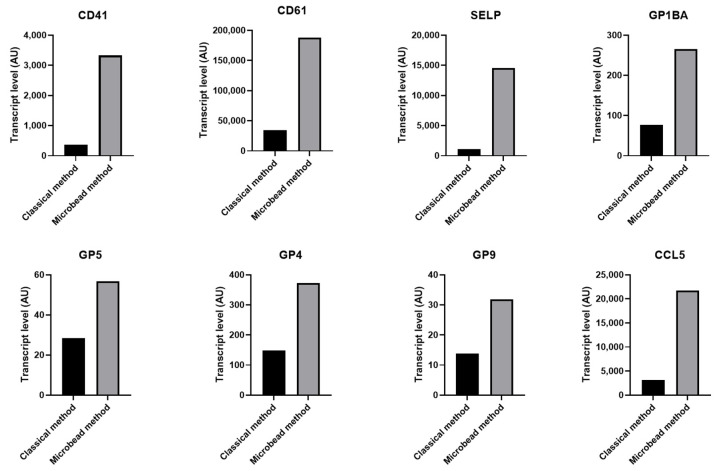
Decreased level of platelet related transcripts in the sample purified without magnetic microbeads. Histograms presenting transcript level for a number of platelet genes, detected by microarray in platelet preparations purified with or without magnetic microbeads.

In platelets collected by the classical method, we observed a lower amount of platelet specific transcripts e.g., ITGA2B (CD41), ITGB3 (CD61), GP1BA, GP1BB, GP9, and GP5, compared to platelets purified using magnetic microbeads. In addition, the relative amount of many platelet-related transcripts e.g., SELP, CCL5, THBS1 CD9, CD36, TGFB, MYL9, and MHC class I and the two Fc receptors FCGR2A and FCER1G were also reduced (Figure 5 and Supplementary Table S4).

## 3. Discussion

In this study, we showed the major impact of leukocytes and erythrocytes contamination on platelet transcriptome and the need to take serious precautions when considering platelet purification for transcriptomic analysis.

Since the discovery of RNAs inside platelets, scientists started to focus on the importance of these RNAs in health and diseases. However, platelets have always been a difficult blood cell to work with in the research setting. They are not easy to manipulate because of their relatively small size, their lack of nucleus and their highly sensitive nature [1]. Platelet preparations obtained by classical purification method are generally contaminated by numbers of leukocytes and erythrocytes [9]. In addition, platelets are usually used in relatively high concentrations, increasing the number of contaminating cells and consequently, platelet transcriptome profile might be significantly altered. In our study, we observed that the relative amount of 8274 transcripts were different between compared samples with a strong leukocyte transcript signature in platelet purified by the classical method. This difference is not due to change in platelets gene expression but reflects the presence or absence of contamination by leucocytes and erythrocytes. We detected transcripts described by Palmer et al. [13] as cell-type specific signature of leukocytes, including transcripts preferentially expressed in B cells (e.g., immunoglobulins and MHC class II molecules), T lymphocytes (e.g., CD3 delta and gamma molecules) and CD8^+^ cytotoxic T cells (e.g., KLRC1). Interestingly, C Palmer et al. also found that transcripts encoding ribosomal proteins, such as RPL23, RPL27, RPL31, RPL35, RPS14, RPS21, RPS3A, and RPS6, were strongly dominant in lymphocyte signature [13]. The relative amount of all of these ribosomal protein encoding transcripts was increased in platelets purified without the use of magnetic microbeads (Supplementary Tables S1 and S5), suggesting that the source of this ribosomal signature is contaminating lymphocytes. In the first comprehensive platelet transcriptome study using RNA-seq, the authors identified 9538 transcripts expressed in human platelets [14]. This list was intersected with our 8274 detected transcripts to determine their overlap (Supplementary Figure S2 and Table S6). Among the 1860 common transcripts, we observed key transcripts related to platelet function such as integrins (ITGA2B, ITGB3), glycoproteins (GP9, GP5, GP1BB, GP1BA), thromboxane A synthase 1 (TBXAS1), and platelet-derived growth factor C (PDGFC). In a number of studies, authors were wary about platelet contamination by leukocytes and used different methods to eliminate this contamination such as filters and magnetic microbeads [12,14,15,16,17,18]. However, in other studies, leukocyte contaminations were not removed and consequently platelet transcriptome profile might be strongly altered [19,20,21]. For example, in Eicher et al. study, where platelets were purified by the classical method, CD45 transcript and a strong ribosomal signature were found in platelet preparations, indicating that transcriptomic results may be altered by the presence of contaminating lymphocytes [19]. Similarly, Raghavachari et al. showed that CD45, identified among platelet-abundant genes, and other leukocyte derived transcripts like CD8 and CD3G were also detected in platelets transcriptome, suggesting an effect of contaminating leukocytes [20]. In a separate study, Healy et al. showed that myeloid-related protein-14 (S100A9) is overexpressed in platelets from patients with acute ST-segment–elevation myocardial infarction (STEMI) compared to patients with stable coronary artery disease (CAD), suggesting a new candidate biomarker for stable coronary artery disease [21]. However, some factors may need to be considered in their study. Beside the use of the classical method to purify platelets, baseline characteristics of patients indicate that STEMI patients have significantly increased leukocyte counts compared to CAD patients and that platelet preparations contained 0.28% leukocytes in STEMI samples and 0.33% leukocytes in CAD samples. Consequently, these low percentages represent a relatively high number of contaminating leukocytes considering the billions of platelets analyzed. Indeed, in our study, where platelets were obtained from a same subject, we also showed that the level of S100A9 transcript was 2.33-fold higher in contaminated platelet preparation (Supplementary Table S1), demonstrating that platelet transcriptome profiles should be interpreted with caution. Recently, platelet transcriptome profiling of 204 healthy donors was performed in order to establish a reference platelet RNA data set for future investigations. In this study, platelet RNA sequencing was performed using a well-established thromboSeq protocol in which platelets were isolated by classical method [22]. Although authors claimed a very low contamination of their samples, many leukocyte-derived transcripts were detected such as CD45, KLRB1, KLRD1, KLRF1, CD3D, CD3E, and IL2RG. Therefore, additional purification step may be required in thromboSeq protocol for future investigations. Importantly, it should be noted that in some studies where microbeads method was used, a number of leukocyte derived transcripts were detected, but their expression levels are low compared to other study in which classical method and the same technique of transcriptome profiling were used [14,18,19]. In addition to microarray analysis and RNA sequencing, qRT-PCR is commonly used in the field of platelet research. In many studies where qRT-PCR was used to assess platelet gene expression levels, platelets were purified by the classical method, raising suspicions that results may be strongly altered by the presence of contaminating cells [23,24,25]. Importantly, platelet contamination by erythrocytes have always been neglected in the majority of platelet transcriptome studies, as mature erythrocytes are anucleate cells containing few RNAs [26]. Yet, our results showed that, although we could not detect the erythrocyte specific transcript CD235a, we found an increased relative amount of hemoglobin α and β chains transcripts in platelets purified by the classical method. In adult humans, two α- and two β-globin chains, each complexed with heme molecule, constitute oxygen-carrying protein called hemoglobin, which is the major protein in erythrocytes [27]. The increased relative amount of α- and β-globin transcripts in contaminated platelets should alert researchers to the need for more precaution in regard of erythrocytes contamination in future investigations. Strikingly, we also observed that in the presence of contaminating cells, the relative amount of specific platelet transcripts, such as CD41 and CD61, were reduced. This could be explained by the normalization method of gene expression levels, such as RMA algorithm which is used in our analysis. RMA algorithm assumes that only few genes are differentially expressed in any compared samples and that the number of overexpressed genes and underexpressed genes are roughly the same [28,29]. In our study, the strong increase of leukocytes- and erythrocytes-derived transcripts in contaminated platelet preparation violated the normalization assumption and the balance between overexpressed and underexpressed genes which cause an over-normalization of the data and produce many falsely reduced transcripts. This distortion of biological signals was also described in microarray analysis of cancer cells in which genes are widely upregulated compared to normal cells producing many falsely down-regulated genes when usual normalization methods were used [28,29].

In conclusion, leukocytes and erythrocytes contamination must be carefully eliminated in every platelet transcriptome analysis, in order to avoid erroneous data interpretation, caused by contaminants derived RNAs.

## 4. Materials and Methods

### 4.1. Study Subjects

Blood samples were obtained from 8 donors for flow cytometry and RT-PCR analysis. Blood from the same donor was used for transcriptome profiling by microarrays. All subjects signed an informed consent form before inclusion in the study. The research was approved by the relevant institutional review boards and ethics committees (n° 2020T2-02 HPS MINEURS (2019-A03054-53) (RCAPHM19_0161) CPP TOURS-Région Centre-Ouest 1). No subject was taking anti-platelet medication.

### 4.2. Platelet Isolation

Blood samples were collected in sodium citrate tubes (BD Vacutainer, Franklin Lakes, NJ, USA) and centrifuged at 200× *g* for 20 min in order to obtain PRP. Platelets were then pelleted by PRP centrifugation at 1000× *g* for 10 min in the presence of 2 mM of ethylenediaminetetraacetic acid (EDTA).

For the “classical method”, platelet pellets were resuspended in cell lysis buffer (RLT buffer added with β-mercaptoethanol, RNeasy Mini Kit, (Qiagen, SAS, France), and stored until RNA extraction.

For the “microbead method”, platelet pellets were resuspended in PBS, 0.5% bovine serum albumin, 2.5 mM of EDTA, and 175 ng·mL^−1^ of prostaglandin E1, and incubated with anti-CD45 and anti-CD235a magnetic microbeads (MiltenyiBiotec, BergischGladbach, Germany) to eliminate residual leukocytes and red blood cells, respectively. Platelets were collected by negative selection using MACS columns (MiltenyiBiotec, BergischGladbach, Germany) and centrifuged at 1000× *g* for 10 min. The last pellet was resuspended in cell lysis buffer and stored until RNA extraction.

### 4.3. RNA Purification

Total RNA was extracted using the RNeasy Mini kit (Qiagen, SAS, France) according to manufacturer indications. RNA concentration and integrity were evaluated using a Nanodrop (ThermoFisher Scientific; Illkirch, France) and an Agilent 2100 Bioanalyzer (Agilent Technologies, Santa Clara, CA, USA), respectively. All RNA samples were stored at −80 °C until the RT-PCR and microarray experiments.

### 4.4. Real-Time Quantitative PCR Analysis

Total RNA was reverse-transcribed into cDNA using QuantiTect Reverse Transcription Kit (Qiagen, SAS, France). Quantitative real-time PCR assays were performed in a LightCycler480 (Roche, Basel, Switzerland), with the use of SybrGreen (Roche Diagnostics, Mannheim, Germany), specific primers (Eurofins MWG Operon, Ebersberg, Germany) for CD45 and CD235a genes and the housekeeping 18S gene as an internal control. Each sample was run in duplicate. The cycling conditions included an initial cycle (95 °C for 10 min) that was followed by 45 cycles (95 °C for 10 s; 60 °C for 20 s; 72 °C for 20 s). The gene expression level was calculated with the 2^−ΔCt^ method. The primer sequences are shown in Table 1.

### 4.5. Flow Cytometry

PRP and platelet preparation depleted of leukocytes and red blood cells were incubated with FITC-conjugated anti-CD45 and APC-conjugated anti-CD235a (MiltenyiBiotec, BergischGladbach, Germany) for 5 min. Samples (50,000 events per sample) were acquired on a BD-AccuriTM C6 flow cytometer (BD Bioscience, San Diego, CA, USA) and analyzed using FlowJo software (FlowJo, LLC, Ashland, OR, USA).

### 4.6. Microarray Analysis

Affymetrix microarrays were processed in the Microarray Core Facility “Transcriptome” of the Institute in Regenerative Medicine and Biotherapy in Montpellier. The GeneChip WT Pico Amplification Kit (Life Technologies SAS) was used to performed sample preparation in accordance with manufacturer’s instructions. Briefly, biotinylated ds cDNA was prepared according to the WT PICO kit from 1 ng of total RNA. Following fragmentation and terminal labeling, 5.5 μg of ds cDNA were hybridized for 16 h at 45 °C and 60 rpm on Clariom D Human chips, as per the manufacturer’s protocol (Affymetrix, Santa Clara, CA, USA # 902922). The arrays were stained using GeneChip Fluidics Station 450. Later, the chips were scanned with GeneChip™ scanner 3000. The DAT files were acquired from the fluorescent signals of the array. Affymetrix GeneChip Command Console (AGCC) software was used for converting the raw data of ARR and DAT image files into intensity data files (.CEL files). Data processing (.CEL files containing intensity data) were analyzed with RMA algorithm at a gene and an exon level and primary analysis were performed using Expression Console Software 1.4.1. The CHP files were transferred to transcriptome analysis console (TAC) version 4.0 (Affymetrix) to analyze the expression pattern of the genes and to construct a scatter plot. The probe sets were annotated using the Affymetrix annotation file from Netaffx (http://www.netaffx.com) (date accessed: 4 June 2021). Different transcript levels with a fold change higher than 2 or lower than −2 were retained. Gene ontology (GO) enrichment analysis was performed using metascape tools [30].

### 4.7. Statistical Analysis

Statistical analyses for RT-PCR and flow cytometry results were performed using GraphPadrism 9 (GraphPad Software, San Diego, CA, USA) applying Mann–Whitney test. *p*-value ≤ 0.05 was considered significant.

## Figures and Tables

**Table 1:** Primer sequences.

Primer	Direction	Sequences (5′ to 3′)
CD45	Forward	ACCAGGAATGGATGTCGCTA
	Reverse	TGGGGCCTGTAAAAGTGTCC
CD235a	Forward	CAAACGGGACACATATGCAG
	Reverse	GTCGGCGAATACCGTAAGAA
18S	Forward	TCAAGAACGAAAGTCGGAGG
	Reverse	CAGCTTTGCAACCATACTCC

## Data Availability

The data are accessible at the gene expression omnibus (GEO) repository (https://www.ncbi.nlm.nih.gov/geo) with the provisional accession series number GSE193664.

## Supplementary Materials

The following supporting information can be downloaded at: https://www.mdpi.com/article/10.3390/ijms23063100/s1.
